# Sonographic Tophi and Inflammation Are Associated With Carotid Atheroma Plaques in Gout

**DOI:** 10.3389/fmed.2021.795984

**Published:** 2021-12-16

**Authors:** Irene Calabuig, Agustín Martínez-Sanchis, Mariano Andrés

**Affiliations:** ^1^Sección de Reumatología, Hospital General Universitario de Alicante-Instituto de Investigación Sanitaria y Biomédica de Alicante (ISABIAL), Alicante, Spain; ^2^Departamento de Medicina Clínica, Universidad Miguel Hernández, Alicante, Spain

**Keywords:** gout, tophi, inflammation, carotid artery diseases, ultrasonography, power Doppler

## Abstract

**Objective:** Gout and cardiovascular disease are closely related, but the mechanism connecting them remains unknown. This study aims to explore whether urate crystal deposits and inflammation (assessed by ultrasound) are associated with carotid atherosclerosis.

**Methods:** We included consecutive patients with crystal-proven gout newly presenting to a tertiary rheumatology unit. Patients under urate-lowering treatment were excluded. Ultrasound assessment was performed during intercritical periods. Musculoskeletal scans evaluated six joints and four tendons for urate crystal deposits (double contour, aggregates, and tophi), and power Doppler (PD) signal (graded 0–3) as a marker of local inflammation. The sum of locations showing deposits or a positive PD signal (≥1) was registered. Carotids were scanned for increased intima-media thickness (IMT) and atheroma plaques, according to the Mannheim consensus. Associations were analyzed using logistic regression.

**Results:** The study included 103 patients showing sonographic crystal deposits at the examined locations (mean sum 9.9, minimum 2); tophi were the most frequent. Two-thirds of participants presented a positive PD signal (30.1% grade 2–3). In the carotid scans, 59.2% of participants showed atheroma plaques, and 33.0% increased IMT. Tophi (odds ratio [OR] 1.24; 95% confidence interval [CI] 1.03–1.50) and a positive PD signal (OR 1.67; 95% CI 1.09–2.56) were significantly associated with atheroma plaques, while an increased IMT showed no sonographic association.

**Conclusion:** Sonographic crystal deposits and subclinical inflammation were consistently observed in patients with intercritical gout. Tophi and a positive PD signal were linked to carotid atherosclerosis. Our findings may contribute to understanding the complex relationship between gout and atherosclerosis.

## Highlights

- All patients with gout showed sonographic crystal deposits in at least two explored locations.
- Subclinical inflammation was also revealed by ultrasound despite performed during the intercritical period.
- A high prevalence of carotid atheroma plaques was confirmed.
- Sonographic tophi and power Doppler signal were associated with carotid atherosclerosis.

## Introduction

Cardiovascular disease (CVD) constitutes the main cause of death worldwide (1) and in patients with inflammatory rheumatic disorders. All-cause mortality is twice as high in people with gout as in the general population (2, 3), mainly due to CVD (4, 5).

For a long time, elevated cardiovascular risk in gout has been attributed to traditional cardiovascular risk factors, which tend to be more prevalent in this group of patients than in the general population (6, 7). However, these risk factors cannot account for all the excess mortality seen, suggesting that gout is associated with additional cardiovascular risk, independent of known determinants of CVD (4, 5, 8, 9). Inflammation from any cause plays a crucial role in the atherogenesis process; not only is it considered an important cardiovascular risk factor by itself (10), it also boosts the effect of other risk factors. In gout, inflammation is stimulated by monosodium urate (MSU) crystals through IL-1β production and neutrophil extracellular traps (NETs) in flares. During intercritical periods, low-grade inflammation persists in peripheral blood (11), synovial fluid (12), and tophi (13), as indicated on ultrasound by a persistent power Doppler (PD) signal and progressive erosions in the absence of flares (14, 15). The formation of NETs (16), along with oxidative stress driven by hyperuricemia (17, 18), damages endothelial cells and leads to endothelial dysfunction, which ultimately results in atherosclerosis.

People with gout show variable levels of cardiovascular risk. Some disease characteristics, such as the presence of tophi, bone erosions, longer duration, oligoarticular or polyarticular presentations, and higher serum urate levels, are predictors of subsequent fatal and non-fatal cardiovascular events (19–21). These are indeed markers of severe disease, with a higher crystal and inflammatory load (11, 22).

Subclinical carotid atherosclerosis, and especially atheroma plaques, entail a very high cardiovascular risk, according to European Society of Cardiology guidelines (23, 24), predicting the development of stroke and coronary heart disease (25, 26). The prevalence of carotid atherosclerosis in people with gout ranges from about 29.1 to 48.9% (27–30) and is likely to be higher than in the non-gouty population. In one study, its identification by ultrasound helped reclassify 27.8% of newly seen gout patients to the very high cardiovascular risk level (30). This reclassification was independent of other cardiovascular risk factors, revealing the poor performance of risk assessment tools such as the Framingham Heart Study (FHS) or the Systematic Coronary Risk Evaluation (SCORE) to predict the presence of carotid atherosclerosis.

Two previous reports failed to demonstrate an association between clinical characteristics of gout and the presence of subclinical atherosclerosis (29, 30). This study aims to explore the association between sonographic signs of MSU crystal deposits and accompanying inflammation, and the presence of carotid atherosclerosis. We hypothesized that a higher sonographic crystal load, depicted as double contour (DC) sign, aggregates, tophi, or higher inflammatory signs, detected by a PD signal, would be associated with the presence of subclinical atherosclerosis in patients with gout.

## Patients and Methods

### Study Design and Population

We designed an observational, prospective study to assess the relationship between carotid atherosclerosis and both sonographic crystal deposits and subclinical articular inflammation, and the impact of a treat-to-crystal dissolution strategy (urate-lowering therapy [ULT], flare prophylaxis) and cardiovascular risk management. Here we present the baseline analysis of the study.

The study took place in a tertiary rheumatology unit of a university hospital, covering a population of 278,095 inhabitants (2019 data). Selection of participants followed a consecutive sampling of incident cases. Consecutive, newly seen patients with crystal-proven gout confirmed in synovial fluid or tophus material and not using ULT at diagnosis were eligible for the study. No exclusion criteria applied; previous use of ULT was accepted. Participants were recruited at the time of a gout flare or after its subsidence, but all cases were scanned during intercritical periods (free of inflammatory signs and symptoms). Flare prophylaxis with low-dose colchicine or other agents such as non-steroidal anti-inflammatory drugs was permitted.

Prior to data collection, investigators explained the aim of the study to eligible patients. According to the Declaration of Helsinki, all participants read the study information sheet and signed informed consent. The local Ethics Committee approved the protocol [PI2018/027].

This article was written in accordance with the STROBE statement (31) and EULAR recommendations for reporting ultrasound studies in rheumatic and musculoskeletal diseases (32).

### Variables

Outcome variables were (a) increased intima-media thickness (IMT, > 0.9 mm, measured by automatic measurement system), and (b) the presence of atheroma plaques. Both variables were assessed following bilateral carotid artery ultrasound, performed according to the Mannheim consensus (33).

The primary explanatory variables were the presence of a DC sign, aggregates, tophi, and PD signal in the ultrasound evaluating MSU crystal deposits (according to OMERACT definitions for MSU crystal deposits and articular inflammation) (34, 35).

Secondary explanatory variables included demographic and clinical characteristics, including cardiovascular risk factors, history of CVD, cardiovascular risk categories, and others involving gout disease and therapeutics (full list of secondary explanatory variables provided in the Supplementary Data 1). These were collected at clinics by attending physicians.

### Ultrasound Assessment

Participants were referred for a musculoskeletal and carotid ultrasound assessment. The two sonographers (AMS and MA) who performed the study had accredited experience in vascular and musculoskeletal ultrasound (AMS: 20 years of experience, level 2 European Federation of Societies for Ultrasound in Medicine and Biology competency, a teacher at the Ultrasound School of the Spanish Society of Rheumatology; MA: 10 years of experience, certified by the Ultrasound School of the Spanish Society of Rheumatology). They were blinded to clinical and laboratory data, and patients were asked not to talk about their disease and treatments. Immediately after ultrasound evaluation, sonographers reported their findings, which were available to the patient and the attending rheumatologist.

The ultrasound system used was the Mindray DC-70 device (Mindray Medical International Ltd, Shenzhen, PRC), with a high frequency (6–14 MHz) linear probe (L14-6NE model) and greyscale and Doppler modalities. Frequencies of at least 12 MHz were used to scan for elementary lesions; PD signal assessment was optimized by adjusting gain, reducing pulse repetition frequency, and placing the scanning box to the region of interest.

**Figure 1:**
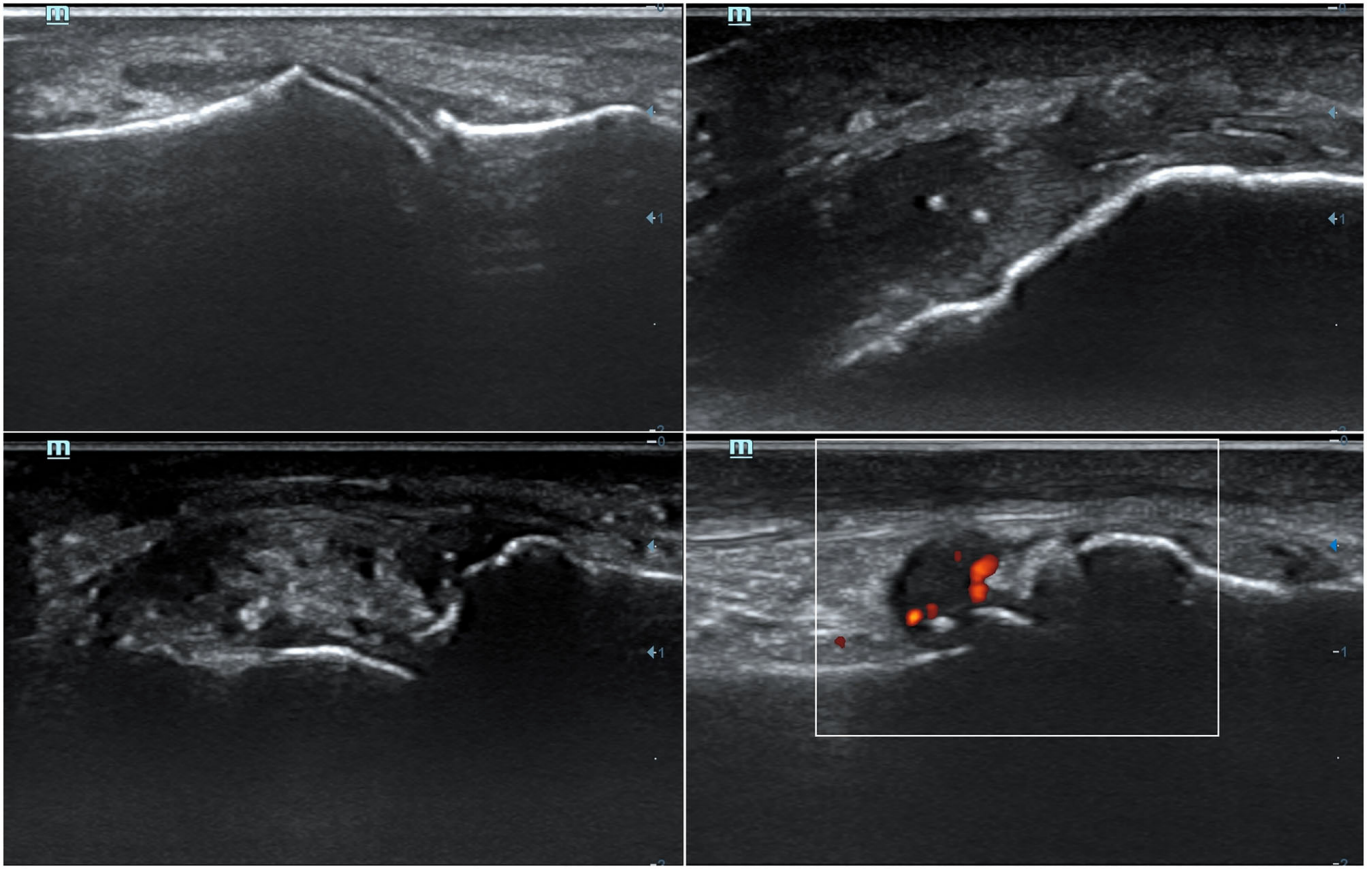
Representative ultrasound features of monosodium urate crystal deposits and associated inflammation. (Up-left) A double contour sign at the cartilage of the 1st metatarsal bone. (Up-right) Two aggregates inside distal patellar tendon. (Bottom-left) A tophus seen at the radial aspect of the 2nd metacarpophalangeal joint. (Bottom-right) A positive (grade 2) power-Doppler signal at 1st metatarsophalangeal joint, indicative of synovitis.

For the musculoskeletal assessment, we followed a binary (presence/absence) scoring system at the anatomical region level, as suggested by Naredo et al. (36). The domains were (a) elementary lesions of MSU crystal deposits (DC sign, aggregates, and tophi), following OMERACT definitions (34) (Figure 1); and (b) inflammation, assessed by the presence of a local PD signal and graded in a semiquantitative score as 0–3 (35) (Figure 1). The evaluated regions, adapted from Naredo et al. (36), were wrists (radiocarpal and midcarpal), second metacarpophalangeal (MCPs) and first metatarsophalangeal (MTPs) joints, and triceps and patellar tendons and their entheses. For upper limb scans, patients remained seated, with their hands over a flat surface for hand scans and with an elbow flexion of 90 degrees for triceps tendon scans. For lower limb scans, patients laid in a supine position, with knee flexion of 90 degrees for patellar tendon and feet scans. All regions were examined longitudinally for the whole width of their dorsal aspects; for second MCPs and first MTPs, medial aspects were also examined.

**Figure 2:**
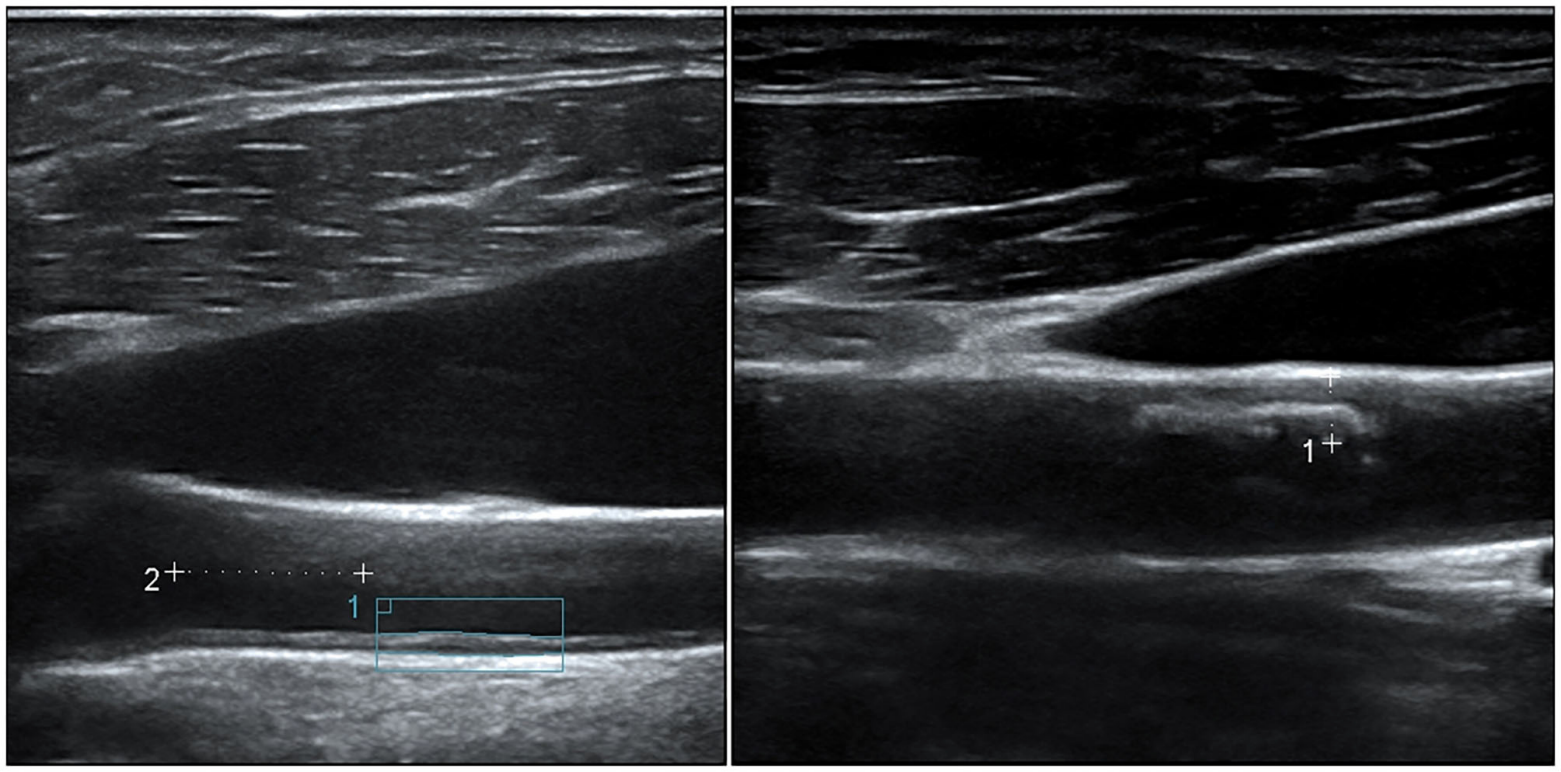
Representative ultrasound features of carotid atherosclerosis. (Left) An increased intima-media thickness (1.048 mm), measured 1 cm proximal to the carotid bulb according to the Mannheim consensus. (Right) A partially calcified atheroma plaque (maximal thickness 3.1 mm) at the anterior wall of the common carotid artery.

Carotid arteries were bilaterally scanned for increased IMT and presence of atheroma plaques, according to the Mannheim consensus (33) (Figure 2). Participants laid in a supine position with a mild hyperextension of the neck. Common carotids and their branches were scanned longitudinally and transversally for plaques, while IMT was measured longitudinally at common carotids.

### Statistical Analysis

#### Sample Size

The prevalence of carotid atheroma plaques ranges between 29.1 and 48.9% (27–30). Assuming a prevalence of 40%, with 80% power, a significance level of 95%, and an estimated rate of refusal to participate of 10%, the estimated sample size was a minimum of 86 patients.

#### Statistical Analysis

Descriptive statistics are presented as frequencies (*n*) and percentages (%) for qualitative variables and as mean (standard deviation) or median (interquartile range) for parametric or non-parametric quantitative variables, respectively. Three quantitative variables were categorized by standard definitions: obesity (body mass index ≥30 kg/m^2^), chronic kidney disease (glomerular filtration rate < 60 mL/min/1.73 m^2^, according to the CKD-EPI equation) and severe chronic kidney disease, stage 4–5 (glomerular filtration rate < 30 mL/min/1.73 m^2^, according to the CKD-EPI equation).

To ensure intra- and interobserver reliability, both sonographers performed the aforementioned musculoskeletal and carotid ultrasound assessment in three patients on the same day and repeated the examination 2 weeks later. Reliability was evaluated by Cohen's κ. The κ for intra-observer agreement ranged from 0.65 to 0.75 (*p* < 0.001); for inter-observer concordance, κ was 0.66 (*p* = 0.001). These results indicate a good level of agreement, similar to the work by Naredo et al. (36).

The sum of locations showing elementary lesions of MSU crystal deposits or positive PD signal (≥1) was estimated to assess crystal and inflammatory burden, respectively. Sum scores for DC sign, aggregates and tophi were given for locations with deposits or a positive PD signal, and individually for each elementary lesion.

The association analysis employed simple logistic regression, considering increased IMT or atheroma plaques as the dependent variables.

A sensitivity analysis was planned, limiting a positive PD signal to scores of 2 or 3, which represent a significant inflammatory burden; a grade 1 PD signal may sometimes be found in healthy individuals (37).

No specific approaches were followed to deal with missing data.

IBM SPSS Statistics for Windows, version 25.0 (IBM Corp., Armonk, N.Y., USA) was used for the analyses.

## Results

### Clinical, Demographic, and Gout Characteristics

A total of 103 patients with crystal-proven gout were included in the study between December 2017 and June 2020; no patients were excluded. Table 1 presents the characteristics of the enrolled participants. Patients were mostly white men, with a mean age of 62 years. At diagnosis, 91 (90.1%) had hyperuricemia (serum urate level ≥ 6.8 mg/dL), and 26 (25.2%) had chronic kidney disease. Twenty-three (22.3%) had established CVD. According to the cardiovascular risk categories, 33% were at moderate risk, 29% at high risk and 38% at very high risk, while none showed a low risk. Regarding gout characteristics, time from the first flare to diagnosis was long (median 4 years), tophi were present in 17 (16.7%), and almost half the patients presented with an oligo- or poly-articular flare, all features indicating a high MSU crystal burden. Ninety patients were taking colchicine at the time of the ultrasound (mode dose of 0.5 mg/day, 17.8% received 1 mg/day).

**Table 1:** Participants' characteristics and comparisons according to carotid ultrasound findings.

		**Increased IMT**			**Atheroma plaque**		
**Variables**	**Total (*n =* 103)**	**Yes (*n =* 33)**	**No (*n =* 67)**	***p***	**Yes (*n =* 61)**	**No (*n =* 42)**	***p***
**Demographic characteristics**							
Age in years, mean (SD)	62.3 (14.1)	70.1 (10.2)	58.3 (14.2)	**<0.001**	68.1 (10.8)	53.9 (14.3)	**<0.001**
Men, *n* (%)	94 (91.3)	31 (93.9)	60 (89.6)	0.471	55 (90.2)	39 (92.9)	0.634
White, *n* (%)	92 (89.3)^*^	31 (93.9)	58 (86.6)	0.268	55 (90.2)	37 (88.1)	0.738
**Clinical characteristics**							
Serum urate (mg/dL), mean (*SD*) [*n = 101*]	8.2 (1.5)	8.1 (1.8)	8.3 (1.3)	0.606	8.2 (1.6)	8.3 (1.4)	0.805
GFR (CKD-EPI, mL/min/1.73 m^2^), mean (*SD*)	75.2 (22.4)	69.5 (18.7)	78.1 (23.5)	0.067	70.6 (22.6)	81.8 (20.7)	**0.012**
CKD (GFR <60 mL/min/1.73 m^2^), *n* (%)	26 (25.2)	11 (33.3)	14 (20.9)	0.177	19 (31.1)	7 (16.7)	0.096
CKD stage 4–5 (GFR <30 mL/min/1.73 m^2^), *n* (%)	3 (2.9)	0 (0.0)	3 (4.5)	0.549	3 (4.9)	0 (0.0)	0.145
LDL-cholesterol (mg/dL), mean (SD)	112.8 (43.4)	111.4 (46.7)	113.6 (49.2)	0.813	110.4 (46.6)	116.3 (38.5)	0.946
BMI (kg/m^2^), mean (SD) [*n = 98*]	30.3 (4.5)	30.4 (4.3)	30.4 (4.7)	0.988	30.4 (4.6)	30.2 (4.5)	0.819
Obesity (BMI ≥ 30), *n* (%) [*n = 98*]	47 (48.0)	16 (53.3)	31 (47.7)	0.609	27 (47.4)	20 (48.8)	0.890
Tobacco consumption, *n* (%) [*n = 101*]	22 (21.8)	7 (21.9)	13 (19.7)	0.802	14 (23.3)	8 (19.5)	0.648
Hypertension, *n* (%)	62 (60.2)	22 (66.7)	38 (56.7)	0.340	41 (67.2)	21 (50.0)	0.079
Diabetes, *n* (%)	23 (22.3)	10 (30.3)	13 (19.4)	0.223	21 (34.4)	2 (4.8)	**<0.001**
Dyslipidemia, *n* (%)	57 (55.3)	21 (63.6)	35 (52.2)	0.280	44 (72.1)	13 (31.8)	**<0.001**
Use of diuretics, *n* (%)	40 (38.8)	17 (51.5)	21 (31.3)	0.051	30 (49.2)	10 (23.8)	**0.009**
Use of lipid-lowering drugs, *n* (%)	37 (35.9)^†^	14 (42.4)	23 (34.3)	0.430	30 (49.2)	7 (16.7)	**0.001**
History of CVD, *n* (%)	23 (22.3)	11 (33.3)	10 (14.9)	**0.034**	19 (31.1)	4 (9.5)	**0.010**
SCORE, mean (*SD*) [*n = 99*]	4.8 (3.9)	7.2 (4.9)	3.6 (2.8)	**<0.001**	6.2 (4.3)	2.8 (2.1)	**<0.001**
FHS, mean (*SD*) [*n = 99*]	6.4 (3.9)	8.6 (4.7)	5.4 (3.0)	**<0.001**	7.8 (3.9)	4.5 (2.9)	**<0.001**
**Gout-related variables**							
Years since the first flare, median (IQR) [*n = 100*]	4.0 (0.0–10.0)	4.0 (0.0–10.0)	4.0 (0.0–10.0)	0.909	5.0 (0.0–11.8)	1.0 (0.0–6.5)	**0.004**
Number of flares, median (IQR) [*N = 100*]	3.0 (1.0–10.0)	2.0 (1.0–5.0)	3.5 (1.0–12.8)	0.054	4.0 (2.0–13.5)	2.0 (1.0–5.0)	**0.009**
Number of involved joints, median (IQR)	2.0 (1.0–5.0)	2.0 (1.0–3.0)	2.0 (1.0–6.0)	0.193	2.5 (1.0–6.0)	2.0 (1.0–3.0)	**0.007**
Presence of tophi, *n* (%) [*N = 102*]	17 (16.7)	5 (15.6)	11 (16.4)	0.920	14 (23.3)	3 (7.1)	**0.031**
**Pattern of last flare**							
Monoarticular, *n* (%)	58 (56.3)	13 (39.4)	42 (62.7)	0.083	29 (47.5)	29 (69.0)	0.094
Oligoarticular, *n* (%)	37 (35.9)	16 (48.5)	21 (31.3)	-	26 (42.6)	11 (26.2)	-
Polyarticular, *n* (%)	8 (7.8)	4 (12.1)	4 (6.0)	-	6 (9.8)	2 (4.8)	-
Prophylaxis at the time of ultrasound, *n* (%)	91 (88.3)^‡^	29 (87.9)	60 (89.6)	0.801	53 (86.9)	38 (90.5)	0.577

*P values were considered significant below 0.050 (bold)*.

* *Ancestries other than white European: Latin American, n = 4 (3.9%); Arabic, n = 4 (3.9%); Roma, n = 2 (1.9%); Asian, n = 1 (1.0%)*.

† *Lipid-lowering drugs: statins, n = 34 (2 in combination with fibrates and 1 with ezetimibe); fibrates in monotherapy, n = 3*.

‡ *Prophylactic agents: colchicine, n = 90 (87.4%); prednisone, n = 1 (1.0%)*.

*IMT, intima-media thickness; SD, standard deviation; GFR, glomerular filtration rate; CKD-EPI, Chronic Kidney Disease Epidemiology Collaboration; CKD, Chronic Kidney Disease; LDL, low-density lipoprotein; BMI, body mass index; CVD, cardiovascular disease; SCORE, Systematic Coronary Risk Evaluation; FHS, Framingham Heart Study; IQR, interquartile range*.

### Ultrasound Assessment

All participants underwent ultrasound assessment, performed a mean of 28.9 days (SD 22.6) after diagnosis. Table 2 shows the extent of the signs of MSU crystal deposit and inflammation. All participants showed sonographic signs of crystal deposits at the examined locations, with a mean sum of 9.9 and a minimum of 2, with tophi standing out as the most frequent. The mean sum of locations with positive PD signal was 1. The rate of patients presenting positive PD signal was 67.0% (grade 2–3 in 30.1%). Positive PD signal significantly correlated with deposits (*r* = +0.37, *p* < 0.001), mainly due to tophi (*r* = +0.37, *p* < 0.001), and aggregates (*r* = +0.20, *p* = 0.040), but not to the DC sign (*r* = +0.12, *p* = 0.232).

**Table 2:** Musculoskeletal ultrasound findings and their association with carotid atherosclerosis.

			**Increased IMT (*****n =*** **33)**		**Atheroma plaque (*****n =*** **61)**	
**Sum of locations with**	**Mean (SD)**	**Range**	**OR (95% CI)**	***p***	**OR (95% CI)**	***p***
Deposits	9.9 (4.1)	2–21	1.06 (0.95–1.17)	0.290	1.07 (0.97–1.19)	0.162
Double contour sign	0.9 (1.0)	0–5	1.05 (0.68–1.61)	0.830	1.03 (0.69–1.56)	0.878
Aggregates	4.1 (2.8)	0–10	1.01 (0.87–1.18)	0.895	1.01 (0.88–1.17)	0.856
Tophi	4.9 (2.3)	0–10	1.19 (0.98–1.44)	0.088	1.24 (1.03–1.50)	**0.026**
Positive PD signal (≥1)	1.1 (1.1)	0–5	0.79 (0.53–1.20)	0.272	1.67 (1.09–2.56)	**0.019**
PD signal 2–3	0.4 (0.7)	0–3	0.85 (0.44–1.64)	0.621	1.70 (0.87–3.33)	0.122

*P values were considered significant below 0.050 (bold)*.

*IMT, intima-media thickness; SD, standard deviation; OR, odds ratio; CI, confidence interval; PD, power Doppler*.

Regarding the carotid scans, atheroma plaques were present in 61 individuals (59.2%) and were found bilaterally in 33 (32.0%). IMT measurement was not possible in three patients due to extensive calcified atherosclerosis. Thirty-three participants showed increased IMT, which was bilateral in 13.

The distribution of musculoskeletal and carotid ultrasound findings across secondary explanatory variables is shown in Table 1 and Supplementary Tables 1, 2.

### Association Analysis

Table 2 presents the results of the logistic regression, assessing the association between carotid ultrasound findings and signs of MSU crystal deposits and inflammation. The extent of tophi and positive PD signal were significantly associated with the presence of atheroma plaques. No association was found with increased IMT.

An analysis of bilateral carotid ultrasound findings (Supplementary Table 3) showed that bilateral atheroma plaques were linked to the extent of locations with deposits, tophi, and positive PD signal. As above, no associations were found with a bilateral increased IMT.

## Discussion

The relationship between gout and CVD has been amply documented, with some studies supporting a causative role for gout. Nonetheless, the mechanism by which these entities are connected is still unknown. In this study, we discovered that MSU crystal deposits and consequent intercritical inflammation are linked with the presence of subclinical carotid atherosclerosis. We found that the extent of sonographic MSU crystal load (mainly by tophi) and inflammatory load expressed as a positive PD signal, were associated with the presence of carotid atheroma plaques. This finding may represent a paradigm shift for gout management. All patients with gout, even those with infrequent flares, showed sonographic signs of MSU crystals, especially tophi. The consistent presence of deposits, along with the deleterious association identified in our study, would merit an early initiation of ULT, even after the first flare, once the disease is definitively diagnosed. An early treatment of gout has indeed demonstrated a reduction of flares and inflammation by imaging (38); this approach would hypothetically reduce the ultimate risk of developing CVD. However, experts still recommend delaying the initiation of ULT until the disease has reached a certain severity (39).

Doppler techniques show the degree of vascularization or blood flow at the examined locations. A positive PD signal translates to inflammation in chronic inflammatory arthritis, and it correlates with the cytokine profile (40) and histopathological synovitis (41). In gout flares, the PD signal is markedly increased; it can persist at lower levels during intercritical periods (14, 42, 43) and decrease during ULT (43). In our series, where two out of three patients showed a positive PD signal (a surrogate marker of crystal-led inflammation), an association was observed with atheroma plaques. Our data thus suggest that the PD signal might be an indicator of cardiovascular risk and a potential target for preventing CVD. In line with our clinical practice, 87% of participants were on prophylactic colchicine initiated after proving the diagnosis of gout, which might have influenced the results due to its anti-inflammatory effect (44). In the study by Peiteado et al. (43), a positive PD signal was found in 96% of patients, with a smaller proportion taking prophylactic colchicine (71%). Thus, our findings should be replicated in a population not under prophylactic agents. Still, the 67% rate of positive PD signal is significant, considering the anti-inflammatory therapeutic background and the intercritical situation of the patients.

Ultrasound in gout has mainly been advocated for diagnosis (45), though some recent papers have proven an interesting role in monitoring the disease, such as checking the reduction of deposits during ULT (46), and predicting the occurrence of flares after discontinuation of colchicine (47) or the achievement of remission criteria in the following 12 months (48). Furthermore, ultrasound could also be of interest for establishing cardiovascular risk. Our results on the extent of tophi and joint inflammation strengthen the value of crystal burden assessment to predict cardiovascular outcomes in patients newly diagnosed with gout. A recent paper from France (49) sustains this point of view. The volume of MSU crystals measured by dual-energy computed tomography predicted subsequent cardiovascular events and mortality in the follow-up. Accordingly, the estimated crystal burden by imaging may even be used, like subcutaneous tophi, to individualize the proper serum urate target for the patient (39).

Our findings support a close relationship between MSU crystals and inflammation, on the one hand, and atherosclerosis on the other. However, the cross-sectional design impedes establishing causality. In addition, all the analyzed variables related to CVD and gout are closely intertwined, as shown in Table 1 and Supplementary Tables 1, 2. Potentially, these issues could have introduced some bias into the association analysis. Larger prospective studies would be desirable, but including groups without ULT is unethical. Nonetheless, the MSU crystal–inflammation–atherosclerosis relationship is supported by the current knowledge available for the atherosclerotic process (10).

This study carries a risk of selection bias, as participants were patients with gout who attended our rheumatology clinic, but not those who are monitored in primary care or those who self-managed their flares. This may have led to a higher rate of comorbidities, cardiovascular risk, crystal burden or a more inflammatory disease.

Women were also underrepresented in our study. Although gout is more prevalent in men, the percentage of female participants (8.7%) is too low. A lower suspicion of gout in women, and accordingly a lower referral to the rheumatology unit, could explain this fact.

## Conclusion

Sonographic deposits of MSU crystals were consistently observed in newly seen patients with gout, and two out of three had inflammation in the intercritical period despite a month on colchicine as average. Crystal and inflammatory load, here shown as tophi and positive PD signal, were found to be associated with carotid atherosclerosis. This new finding may contribute to understanding the complex relationship between gout and atherosclerosis.

## Data Availability Statement

The raw data supporting the conclusions of this article will be made available by the authors, without undue reservation.

## Ethics Statement

The studies involving human participants were reviewed and approved by Alicante General University Hospital, Institute of Sanitary and Biomedical Research (ref. PI2018/027). The patients/participants provided their written informed consent to participate in this study.

## Author Contributions

IC: data curation, investigation, project administration, visualization, writing—original draft, and writing—review and editing. AM-S: funding acquisition, investigation, and writing—review and editing. MA: conceptualization, formal analysis, funding acquisition, investigation, methodology, supervision, visualization, and writing—review and editing. All authors contributed to the article and approved the submitted version.

## Funding

This work was supported by the Spanish Foundation of Rheumatology (Grant exp. 17/EM253/0201). The funder had no role on the study design, data analysis or results communication.

## Conflict of Interest

MA declares consultancies and speaking fees from Menarini and Grünenthal. The remaining authors declare that the research was conducted in the absence of any commercial or financial relationships that could be construed as a potential conflict of interest.

## Publisher's Note

All claims expressed in this article are solely those of the authors and do not necessarily represent those of their affiliated organizations, or those of the publisher, the editors and the reviewers. Any product that may be evaluated in this article, or claim that may be made by its manufacturer, is not guaranteed or endorsed by the publisher.
