# Supplementary material for: Sonographic Tophi and Inflammation Are Associated With Carotid Atheroma Plaques in Gout
Source: Front Med (Lausanne). 2021 Dec 16;8:795984. doi: 10.3389/fmed.2021.795984 (PMC8716736; doi:10.3389/fmed.2021.795984)
Supplement: Supplementary file 1 [file Data_Sheet_1.docx]

**MANUSCRIPT TITLE**

**Sonographic tophi and inflammation are associated with carotid atheroma plaques in gout.**

**SUPPLEMENTARY MATERIAL**

**Supplementary Data S1.** Secondary explanatory variables

Demographic characteristics

- Age (years)
- Gender
- Ancestry: white European, Latin American, Arabic, Roma, Asian or Black

Clinical characteristics

- Serum urate level (mg/dL) at the time of diagnosis
- Glomerular filtration rate (GFR, in mL/min/1.73m^2^, defined by CKD-EPI equation) at the time of diagnosis
- Chronic kidney disease (CKD, if GFR < 60 mL/min/1.73m^2^)
- Chronic kidney disease stage 4-5 (if GFR <30 mL/min/1.73m^2^)
- Low-density lipoprotein level (mg/dL) at the time of diagnosis
- Body mass index (BMI, kg/m^2^) at the time of diagnosis
- Obesity (BMI ≥30)
- Tobacco consumption (by anamnesis)
- Hypertension (clinical diagnosis in patients’ record)
- Diabetes (clinical diagnosis in patients’ record)
- Dyslipidemia (clinical diagnosis in patients’ record)
- Use of diuretics that increase serum urate concentrations: loop diuretics and thiazides
- Use of lipid-lowering drugs: statins, fibrates or ezetimibe
- Background of cardiovascular disease: coronary artery disease, congestive heart failure, cerebrovascular disease or peripheral artery disease including aortic aneurism (in patients’ record)
- Systematic Coronary Risk Evaluation (SCORE)
- Framingham Heart Study or FHS: Spanish adaptation or REGICOR
- Cardiovascular risk categories according to the 2019 European Society of Cardiology/European Atherosclerosis Society Guidelines for the management of dyslipidaemias (doi: 10.1093/eurheartj/ehz826)

Gout characteristics

- Years since the first flare to diagnosis (by anamnesis)
- Number of flares (by anamnesis)
- Number of involved joints (by anamnesis)
- Presence of subcutaneous tophi (by physical exam)
- Pattern of last flare: monoarticular, oligoarticular or polyarticular (by physical exam)
- Use of flare prophylaxis at the time of ultrasound, type (colchicine, non-steroidal anti-inflammatory drugs or prednisone) and dose (mg/day)

**Supplementary Table S1.** Correlation between musculoskeletal ultrasound findings and quantitative secondary variables

|  | **Sum of locations with:** | | | | | | | | | | | |
| --- | --- | --- | --- | --- | --- | --- | --- | --- | --- | --- | --- | --- |
|  | **Deposits** | | **DC sign** | | **Aggregates** | | **Tophi** | | **Positive PD signal (≥1)** | | **PD signal grade 2-3** | |
|  | **r** | **p** | **r** | **p** | **r** | **p** | **r** | **p** | **r** | **p** | **r** | **p** |
| **DEMOGRAPHIC CHARACTERISTICS** |  |  |  |  |  |  |  |  |  |  |  |  |
| Age (years) | 0.008 | 0.937 | 0.002 | 0.986 | −0.133 | 0.181 | 0.176 | 0.076 | 0.222 | **0.024** | 0.040 | 0.688 |
| **CLINICAL CHARACTERISTICS** |  |  |  |  |  |  |  |  |  |  |  |  |
| Serum urate (mg/dL) | −0.051 | 0.616 | 0.122 | 0.225 | −0.120 | 0.230 | 0.004 | 0.965 | 0.168 | 0.093 | 0.097 | 0.334 |
| GFR (CKD-EPI equation, ml/min/1.73m^2^) | 0.070 | 0.483 | 0.069 | 0.489 | 0.142 | 0.152 | −0.076 | 0.448 | −0.116 | 0.244 | 0.064 | 0.521 |
| LDL-cholesterol (mg/dL) | 0.059 | 0.551 | 0.089 | 0.372 | 0.081 | 0.419 | −0.028 | 0.778 | −0.320 | **0.001** | −0.096 | 0.337 |
| BMI (kg/m^2^) | 0.154 | 0.129 | −0.151 | 0.139 | −0.031 | 0.760 | 0.379 | **<0.001** | 0.116 | 0.253 | 0.209 | **0.039** |
| SCORE | 0.090 | 0.376 | 0.040 | 0.694 | −0.010 | 0.920 | 0.161 | 0.110 | −0.008 | 0.937 | 0.006 | 0.950 |
| FHS | 0.226 | **0.025** | −0.013 | 0.898 | 0.176 | 0.082 | 0.206 | **0.041** | −0.009 | 0.933 | 0.069 | 0.946 |
| **GOUT CHARACTERISTICS** |  |  |  |  |  |  |  |  |  |  |  |  |
| Years since the first flare | 0.199 | **0.047** | 0.060 | 0.555 | 0.132 | 0.189 | 0.178 | 0.076 | 0.135 | 0.181 | 0.155 | 0.124 |
| Number of flares | 0.060 | 0.553 | 0.140 | 0.165 | −0.016 | 0.872 | 0.071 | 0.481 | 0.092 | 0.364 | 0.266 | **0.007** |
| Number of involved joints | 0.189 | 0.056 | 0.310 | **0.001** | −0.011 | 0.912 | 0.225 | **0.022** | 0.253 | **0.010** | 0.226 | **0.022** |

P values were considered significant below 0.050 (bold).

Abbreviations: DC: double contour; PD: power-Doppler; r: Pearson’s correlation coefficient; GFR: glomerular filtration rate; CKD-EPI: Chronic Kidney Disease Epidemiology Collaboration; LDL: low-density lipoprotein; BMI: body mass index; SCORE: Systematic Coronary Risk Evaluation; FHS: Framingham Heart Study.

**Supplementary Table S2.** Distribution of musculoskeletal ultrasound findings across qualitative secondary variables, expressed as median (IQR)

|  | **Deposits** | **DC sign** | **Aggregates** | **Tophi** | **Positive PD signal (≥1)** | **PD signal grade 2-3** |  |
| --- | --- | --- | --- | --- | --- | --- | --- |
| **DEMOGRAPHIC CHARACTERISTICS** | | | | | | | |
| **Gender** |  |  |  |  |  |  |  |
| Women | 6.0 (5.0-9.0) | 0.0 (0.0-1.0) | 2.0 (1.0-3.0) | 4.0 (4.0-5.0) | 1.0 (1.0-1.0) | 0.0 (0.0-0.0) |  |
| Men | 10.0 (7.0-13.0) | 1.0 (0.0-2.0) | 4.0 (2.0-7.0) | 5.0 (3.0-6.0) | 1.0 (0.0-2.0) | 0.0 (0.0-1.0) |  |
| *P value* | ***0.023*** | *0.096* | ***0.038*** | *0.475* | *0.715* | ***0.043*** |  |
| **White European** | | | |  |  |  |  |
| No | 9.0 (7.0-13.0) | 1.0 (1.0-2.0) | 4.0 (2.0-6.0) | 4.0 (4.0-6.0) | 1.0 (0.0-1.0) | 0.0 (0.0-0.0) |  |
| Yes | 10.0 (6.5-12.0) | 1.0 (0.0-1.0) | 3.5 (2.0-6.5) | 5.0 (3.0-6.0) | 1.0 (0.0-2.0) | 0.0 (0.0-1.0) |  |
| *P value* | *0.953* | *0.216* | *0.901* | *0.538* | *0.688* | *0.104* |  |
| **CLINICAL CHARACTERISTICS** | | | | | | | |
| **CKD (GFR <60)** |  |  |  |  |  |  |  |
| No | 10.0 (7.0-13.0) | 1.0 (0.0-2.0) | 4.0 (2.0-7.0) | 5.0 (3.0-6.0) | 1.0 (0.0-2.0) | 0.0 (0.0-1.0) |  |
| Yes | 9.0 (7.0-12.0) | 1.0 (0.0-1.0) | 3.0 (2.0-5.0) | 5.5 (4.0-6.0) | 1.0 (1.0-1.0) | 0.0 (0.0-0.0) |  |
| *P value* | *0.449* | *0.383* | *0.260* | *0.631* | *0.465* | *0.342* |  |
| **CKD stage 4-5 (GFR <30)** | | | | | | | |
| No | 9.5 (7.0-12.0) | 1.0 (0.0-1.5) | 4.0 (2.0-6.0) | 5.0 (3.0-6.0) | 1.0 (0.0-2.0) | 0.0 (0.0-1.0) |  |
| Yes | 9.0 (6.0-16.0) | 0.0 (0.0-1.0) | 5.0 (2.0-8.0) | 4.0 (3.0-8.0) | 1.0 (1.0-3.0) | 0.0 (0.0-0.0) |  |
| *P value* | *0.949* | *0.309* | *0.556* | *1.000* | *0.338* | *0.379* |  |
| **Obesity (BMI ≥30)** | | | | | | | |
| No | 9.0 (6.0-12.0) | 1.0 (0.0-2.0) | 4.0 (2.0-7.0) | 4.0 (3.0-6.0) | 1.0 (0.0-1.0) | 0.0 (0.0-1.0) |  |
| Yes | 9.0 (8.0-12.0) | 1.0 (0.0-1.0) | 3.0 (2.0-6.0) | 6.0 (4.0-7.0) | 1.0 (0.0-2.0) | 0.0 (0.0-1.0) |  |
| *P value* | *0.369* | *0.439* | *0.300* | ***0.002*** | *0.385* | *0.418* |  |
| **Tobacco consumption** | | | | | | | |
| No | 10.0 (7.0-13.0) | 1.0 (0.0-1.0) | 4.0 (2.0-7.0) | 5.0 (3.0-7.0) | 1.0 (0.0-2.0) | 0.0 (0.0-1.0) |  |
| Yes | 8.5 (6.0-11.0) | 1.0 (0.0-2.0) | 2.5 (1.0-5.0) | 5.0 (3.0-6.0) | 1.0 (0.0-2.0) | 0.0 (0.0-1.0) |  |
| *P value* | *0.126* | *0.707* | *0.095* | *0.454* | *0.955* | *0.861* |  |
| **Hypertension** |  |  |  |  |  |  |  |
| No | 9.0 (8.0-12.0) | 1.0 (0.0-2.0) | 4.0 (2.0-7.0) | 4.0 (3.0-6.0) | 1.0 (0.0-1.0) | 0.0 (0.0-1.0) |  |
| Yes | 9.5 (6.0-12.0) | 1.0 (0.0-1.0) | 3.0 (2.0-6.0) | 5.0 (4.0-6.0) | 1.0 (0.0-2.0) | 0.0 (0.0-1.0) |  |
| *P value* | *0.600* | *0.352* | *0.203* | *0.511* | *0.315* | *0.411* |  |
| **Diabetes** |  |  |  |  |  |  |  |
| No | 9.0 (6.5-12.0) | 1.0 (0.0-2.0) | 4.0 (2.0-6.0) | 5.0 (3.0-6.0) | 1.0 (0.0-1.5) | 0.0 (0.0-1.0) |  |
| Yes | 10.0 (7.0-14.0) | 1.0 (0.0-1.0) | 3.0 (2.0-8.0) | 6.0 (4.0-7.0) | 1.0 (0.0-2.0) | 0.0 (0.0-1.0) |  |
| *P value* | *0.384* | *0.175* | *0.716* | *0.115* | *0.737* | *0.749* |  |
| **Dyslipidemia** |  |  |  |  |  |  |  |
| No | 9.0 (7.0-12.0) | 1.0 (0.0-1.0) | 3.0 (2.0-6.0) | 5.0 (3.0-6.0) | 1.0 (0.0-1.0) | 0.0 (0.0-0.0) |  |
| Yes | 10.0 (7.0-13.0) | 1.0 (0.0-1.0) | 4.0 (2.0-6.0) | 5.0 (4.0-6.0) | 1.0 (1.0-2.0) | 0.0 (0.0-1.0) |  |
| *P value* | *0.405* | *0.521* | *0.372* | *0.479* | ***0.024*** | *0.159* |  |
| **Use of diuretics** |  |  |  |  |  |  |  |
| No | 10.0 (7.0-12.0) | 1.0 (0.0-1.0) | 4.0 (2.0-7.0) | 5.0 (3.0-6.0) | 1.0 (0.0-2.0) | 0.0 (0.0-1.0) |  |
| Yes | 9.0 (6.5-12.0) | 1.0 (0.0-1.5) | 3.0 (2.0-5.0) | 5.5 (3.5-6.0) | 1.0 (0.5-1.0) | 0.0 (0.0-0.5) |  |
| *P value* | *0.644* | *0.310* | *0.299* | *0.314* | *0.576* | *0.434* |  |
| **Use of lipid-lowering drugs** | | | | | | | |
| No | 9.0 (7.0-12.0) | 1.0 (0.0-2.0) | 3.0 (2.0-6.0) | 5.0 (3.0-6.0) | 1.0 (0.0-1.0) | 0.0 (0.0-1.0) |  |
| Yes | 11.0 (6.0-13.0) | 1.0 (0.0-1.0) | 4.0 (2.0-6.0) | 5.0 (4.0-7.0) | 1.0 (1.0-2.0) | 0.0 (0.0-1.0) |  |
| *P value* | *0.396* | *0.619* | *0.339* | *0.483* | ***0.021*** | *0.162* |  |
| **History of cardiovascular disease** | | | | | | | |
| No | 9.0 (6.0-12.0) | 1.0 (0.0-1.0) | 3.5 (2.0-6.0) | 5.0 (3.0-6.0) | 1.0 (0.0-1.0) | 0.0 (0.0-1.0) |  |
| Yes | 11.0 (8.0-13.0) | 1.0 (0.0-2.0) | 4.0 (2.0-6.0) | 6.0 (4.0-7.0) | 1.0 (1.0-2.0) | 0.0 (0.0-1.0) |  |
| *P value* | *0.217* | *0.827* | *0.520* | *0.075* | *0.081* | *0.906* |  |
| **GOUT CHARACTERISTICS** | | | | | | | |
| **Presence of tophi** | | | |  |  |  |  |
| No | 9.0 (7.0-12.0) | 1.0 (0.0-1.0) | 4.0 (2.0-6.0) | 5.0 (3.0-6.0) | 1.0 (0.0-1.0) | 0.0 (0.0-0.0) |  |
| Yes | 10.0 (6.0-13.0) | 1.0 (1.0-2.0) | 2.0 (1.0-5.0) | 6.0 (4.0-7.0) | 2.0 (1.0-3.0) | 1.0 (0.0-1.0) |  |
| *P value* | *0.614* | ***0.019*** | *0.100* | *0.090* | ***0.009*** | ***0.008*** |  |
| **Pattern of last flare** | | | | | | | |
| Monoarticular | 8.0 (6.0-11.0) | 1.0 (0.0-1.0) | 3.0 (2.0-6.0) | 4.0 (3.0-6.0) | 1.0 (0.0-1.0) | 0.0 (0.0-1.0) |  |
| Oligoarticular | 11.0 (8.0-15.0) | 1.0 (0.0-2.0) | 5.0 (2.0-7.0) | 6.0 (4.0-7.0) | 1.0 (0.0-2.0) | 0.0 (0.0-1.0) |  |
| Polyarticular | 12.0 (11.0-12.5) | 1.5 (1.0-2.5) | 5.0 (2.5-5.0) | 6.0 (4.5-7.5) | 2.0 (1.0-3.0) | 1.0 (0.0-1.0) |  |
| *P value* | ***0.001*** | ***0.009*** | *0.165* | ***0.004*** | *0.094* | *0.122* |  |
| **Prophylaxis at the time of ultrasound** | | | | | | | |
| No | 10.0 (8.5-11.5) | 0.0 (0.0-1.0) | 5.0 (2.0-7.5) | 4.5 (3.0-6.0) | 1.0 (0.5-2.0) | 0.0 (0.0-0.5) |  |
| Yes | 9.0 (6.0-12.0) | 1.0 (0.0-2.0) | 3.0 (2.0-6.0) | 5.0 (3.0-6.0) | 1.0 (0.0-2.0) | 0.0 (0.0-1.0) |  |
| *P value* | *0.654* | *0.186* | *0.188* | *0.400* | *0.572* | *0.725* |  |

P values were considered significant below 0.050 (bold).

Abbreviations: DC: double contour; PD: power-Doppler; IQR: interquartile range; CKD-EPI: Chronic Kidney Disease Epidemiology Collaboration; GFR: glomerular filtration rate; BMI: body mass index.

**Supplementary Table S3.** Musculoskeletal ultrasound findings and their association with bilateral carotid atherosclerosis

|  | **Bilateral increased IMT (n=13)** | | **Bilateral atheroma plaque (n=33)** | |
| --- | --- | --- | --- | --- |
| **Sum of locations with:** | **OR (95%CI)** | **p value** | **OR (95%CI)** | **p value** |
| Deposits | 1.04 (0.91-1.20) | 0.582 | 1.13 (1.02-1.26) | **0.025** |
| Double contour sign | 1.21 (0.69-2.13) | 0.504 | 1.18 (0.78-1.80) | 0.437 |
| Hyperechoic aggregates | 0.99 (0.80-1.23) | 0.941 | 1.06 (0.92-1.24) | 0.420 |
| Tophi | 1.12 (0.86-1.46) | 0.408 | 1.34 (1.09-1.65) | **0.006** |
| Positive PD signal (≥1) | 0.46 (0.21-1.04) | 0.061 | 1.55 (1.05-2.28) | **0.027** |
| PD signal 2-3 | 0.61 (0.19-1.90) | 0.389 | 1.48 (0.82-2.68) | 0.196 |

P values were considered significant below 0.050 (bold).

Abbreviations: IMT: intima-media thickness; SD: standard deviation; OR: odds ratio; CI: confidence interval; PD: power-Doppler.
